# Modeling and Compensating Temperature-Dependent Non-Uniformity Noise in IR Microbolometer Cameras

**DOI:** 10.3390/s16071121

**Published:** 2016-07-19

**Authors:** Alejandro Wolf, Jorge E. Pezoa, Miguel Figueroa

**Affiliations:** 1 Electrical Engineering Department, Universidad de Concepción, Edmundo Larenas 219, Concepción 4030000, Chile; alejandrowolf@udec.cl (A.W.); jpezoa@udec.cl (J.E.P.); 2 Center for Optics and Photonics, Universidad de Concepción, Avda. Esteban S. Iturra s/n, Concepción 4030000, Chile

**Keywords:** physical sensors, imaging, infrared imaging, image enhancement, noise in imaging systems, image reconstruction techniques

## Abstract

Images rendered by uncooled microbolometer-based infrared (IR) cameras are severely degraded by the spatial non-uniformity (NU) noise. The NU noise imposes a fixed-pattern over the true images, and the intensity of the pattern changes with time due to the temperature instability of such cameras. In this paper, we present a novel model and a compensation algorithm for the spatial NU noise and its temperature-dependent variations. The model separates the NU noise into two components: a constant term, which corresponds to a set of NU parameters determining the spatial structure of the noise, and a dynamic term, which scales linearly with the fluctuations of the temperature surrounding the array of microbolometers. We use a black-body radiator and samples of the temperature surrounding the IR array to offline characterize both the constant and the temperature-dependent NU noise parameters. Next, the temperature-dependent variations are estimated online using both a spatially uniform Hammerstein-Wiener estimator and a pixelwise least mean squares (LMS) estimator. We compensate for the NU noise in IR images from two long-wave IR cameras. Results show an excellent NU correction performance and a root mean square error of less than 0.25 ${}^{\circ }$C, when the array’s temperature varies by approximately 15 ${}^{\circ }$C.

## 1. Introduction

Microbolometers are a type of infrared (IR) radiation detector developed by Honeywell in the 80s, to be used as focal plane arrays (FPAs) in imaging systems [1]. Microbolometers are thermal IR sensors, that is, they modify their temperature according to the absorbed IR radiation. The bolometer feature of these sensors means that temperature variations in the sensor produce changes in its electrical resistance. Such a change can be, in turn, measured by means of simple electrical circuits. Finally, the changes in the electrical resistance are processed in a digital fashion to ultimately obtain read-out values quantifying the amount of IR radiation absorbed by the detector. Nowadays, IR cameras based on microbolometers are the most used technology for sensing in the band of 7 to 14 μm, since such technology provides a good enough sensitivity to the applications, at an affordable price. Moreover, unlike other technologies such as HdCdTe, microbolometers do not require cryogenic cooling systems to operate in the long-wave infrared (LWIR) band [2].

It has been observed in the literature that images rendered by uncooled microbolometer-based IR cameras are severely degraded by the spatial non-uniformity (NU) noise, also termed as fixed-pattern noise (FPN). Such noise imposes a fixed-pattern over the true images, and is defined as the spatially heterogeneous response of the IR camera to a uniform incoming radiation [3]. The NU noise is caused by gradients in the fabrication of the IR FPA and readout circuitry, initial conditions of the detectors and temperature gradients [4]. The spatial structure of the NU noise is fixed; however, its intensity varies with time due to the temperature instability of the camera [3,5]. The NU noise is particularly severe in microbolometer-based IR cameras, because temperature variations in the IR detector result in thermal drift and exacerbate the negative effects of the NU noise. Such variations can be produced by either the surrounding environment or internal camera elements such as the lens, the FPA, and the read-out electronics [6,7,8]. Since variations in the camera temperature are unavoidable because they are part of its normal operation, manufactures and users must deal with the problem of compensating the read-out data prior to obtain an accurate measurement of the target of interest.

The temperature-dependent non-uniformity correction (NUC) problem in microbolometer-based IR cameras has traditionally been solved by performing periodic multi-point calibration (MPC) [9], or using adaptive signal-processing-based NUC algorithms based on the statistics of the scene [10,11,12,13,14]. MPC must be performed periodically to maintain radiometric calibration [15]. This requires taking the camera offline and using external temperature sources, which can be impractical for many applications. Otherwise, adaptive signal-processing methods typically improve the visual quality of the image but are unable to achieve radiometric precision. Moreover, they are prone to introduce ghosting artifacts in the corrected images due to overlearning when the raw imagery lacks temporal diversity.

Currently, researchers are attempting to solve the temperature-dependent NUC problem using temperature samples of either the FPA or its surroundings. Nugent et al. proposed stabilizing the raw output of a LWIR camera using a two-parameter first-order function, which requires measuring the temperature difference of the camera between its current operating point and a predefined reference [15]. Krupiński et al., introduced, in uncooled LWIR cameras, additional shutters and modeled the relationship between the FPA temperature and its readout raw response [16]. Cao et al., characterized the microbolometer array using a third-order polynomial that relates the offset of each pixel in the array to the FPA temperature [8]. Krupiński et al., recently proposed a method that uses a coefficient table to compensate for the errors caused by temperature changes in the optics of an IR camera, and computes the error caused by such variations using the camera temperature as an input [17]. Also, in earlier works we modeled the effect of changes in the internal temperature of an IR camera, compensated their effect on the raw images, and implemented compensation algorithms in software and hardware architectures [18,19]. In [20], Olbrycht and Więcek proposed the use of a semi-transparent shutter that is periodically placed in front of the FPA in order to compensate for the thermal drift of the camera. All the aforementioned methods do not exhibit ghosting artifacts and achieve better results than traditional NUC techniques; however, they demand an extensive characterization of the IR camera, frequently use complex models that must be evaluated at run time, and require samples of the FPA temperature.

In this paper, we present a novel model for the spatial NU noise and the raw output of uncooled microbolometer-based IR cameras. In addition, we present an algorithm that compensates for the temperature-dependent variations in the NU noise. The model regards the noise as three independent terms: (i) a time-constant, spatially heterogeneous component, which defines the spatial structure of the FPN; (ii) a temperature-dependent, spatially heterogeneous component, which depends linearly on the FPA temperature fluctuations; and (iii) a temperature-dependent, spatially homogeneous component accounting for the IR radiation generated in the camera. The compensation algorithm exploits this separation to estimate the NU components offline and online. To estimate the spatially heterogeneous noise components at each pixel in the FPA, we use an offline MPC method and vary the temperature of the FPA during calibration. Next, we estimate the temperature-dependent, spatially homogeneous component using a Hammerstein-Wiener (HW) model designed offline, and samples from the temperature sensor. Finally, the temperature-dependent, spatially nonuniform component is estimated using a least mean squares (LMS) estimator and the IR video data.

The remaining part of this paper is organized as follows. In Section 2.1, we derive the model proposed in this work by analyzing the internal and external sources affecting the microbolometer temperature in an IR camera. Next, in Section 2.2, we present the image compensation model, the NU noise parameter estimator as well as the HW model used to track the temperature variations in the IR array. In Section 3, we present test results using two long-wave IR cameras, a commercial a-Si–based camera and a VOx-based camera core, with a black body calibrator and natural IR images. In the same section, the NUC performance of the algorithm is also tested using real IR images. Finally, in Section 4, the main conclusions of our work are presented and the future work is outlined.

## 2. Methodology

### 2.1. A Temperature-Dependent Model for the NU Noise

The model for the spatial NU noise, in uncooled microbolometer-based IR cameras with M×N pixels, can be derived from basic principles. Indeed, the electric response or measured voltage, $Y^{i,j}\left[k\right]$, at the (i,j)th pixel and at the *k*th sample time, in a microbolometer array is given by:

(1) $$\begin{array}{c} Y^{i,j}\left[k\right]=I^{i,j}\left[k\right]R^{i,j}\left[k\right]+V_{elec}^{i,j}\left[k\right] \end{array}$$

where $I^{i,j}\left[k\right]$ is the bias current, $R^{i,j}\left[k\right]$ is the microbolometer resistance, and $V_{elec}^{i,j}\left[k\right]$ is the electric response to resistive elements that are external to the microbolometer itself [5]. For simplicity, we assume both that the latter term is negligible and that the bias current is constant, in time, when the power supply is stable [5].

**Figure 1:**
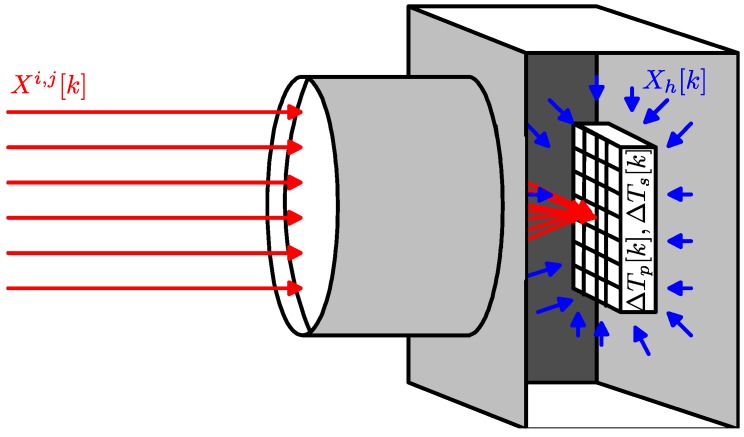
Internal and external sources affecting the microbolometer temperature in an infrared (IR) camera.

The microbolometer resistance, at each pixel in the FPA, varies with time depending on its temperature which, in turn, changes due to different temperature sources. As depicted in Figure 1, we assume that the sources changing the microbolometer temperature are: (i) the measured incident IR irradiance, $X^{i,j}\left[k\right]$; (ii) the IR irradiance generated in the housing of the IR camera, $X_{h}\left[k\right]$; (iii) the temperature changes due to power dissipation, $\Delta T_{p}\left[k\right]$; and (iv) the variation in the surrounding temperature, $\Delta T_{s}\left[k\right]$. Assuming also linear superposition, the microbolometer resistance can be expressed as the microbolometer resistance at k=0, $R_{0}$, plus its changes due to the aforementioned sources:

(2) $$\begin{array}{cc} R^{i,j}\left[k\right] & =R_{0}^{i,j}+\Delta R^{i,j}\left(X^{i,j}\left[k\right]\right)+\Delta R^{i,j}\left(X_{h}\left[k\right]\right)+\Delta R^{i,j}\left(\Delta T_{p}\left[k\right]\right)+\Delta R^{i,j}\left(\Delta T_{s}\left[k\right]\right) \end{array}$$

where the notation ΔR(·) emphasizes the idea of a change in the microbolometer resistance.

The variations in the microbolometer resistance due to temperature changes can be linearly approximated as:

(3) $$\begin{array}{c} \Delta R^{i,j}\left(\Delta T^{i,j}\right)\approx \alpha^{i,j}R_{0}^{i,j}\Delta T^{i,j} \end{array}$$

where $\alpha^{i,j}$ is the thermal resistance coefficient (TRC) and $\Delta T^{i,j}$ is the temperature variation with respect to the temperature of the microbolometer at k=0 [21]. In addition, variations in the microbolometer resistance due to any IR flux can be determined using the heat balance differential equation relating the incident radiation and heat losses [21], and Equation (2). Therefore, the resistance variation caused by the incident IR irradiance and the irradiance generated in the camera housing can be written as:

(4) $$\begin{array}{c} \Delta R^{i,j}\left(X^{i,j}\left[k\right]\right)+\Delta R^{i,j}\left(X_{h}\left[k\right]\right)\approx \frac{\alpha^{i,j}R_{0}^{i,j}}{\beta G^{i,j}}\left(X^{i,j}\left[k\right]+X_{h}\left[k\right]\right) \end{array}$$

where $G^{i,j}$ is the thermal conductivity and *β* is a scale constant.

Plugging in Equations (2)–(4) into (1), we obtain our proposed model for the NU noise and the compensated response, at each pixel, of the microbolometer-based IR camera:

(5) $$\begin{array}{c} X^{i,j}\left[k\right]=S_{0}^{i,j}Y^{i,j}\left[k\right]-S_{1}^{i,j}-S_{2}^{i,j}\left(\Delta T_{p}^{i,j}\left[k\right]+\Delta T_{s}^{i,j}\left[k\right]\right)-X_{h}\left[k\right] \end{array}$$

with $S_{0}^{i,j}=\beta G^{i,j}/I^{i,j}R_{0}^{i,j}\alpha^{i,j}$, $S_{1}^{i,j}=\beta G^{i,j}/\alpha^{i,j}$, and $S_{2}^{i,j}=\beta G^{i,j}$.

Now we comment on the model presented in Equation (5). First, note that the NU noise, at each pixel, is defined by the three $S_{k}^{i,j}$ parameters. Second, the $S_{0}^{i,j}$ and $S_{1}^{i,j}$ parameters are akin to the pixelwise gain and offset parameters commonly used to model the temperature-independent NU noise, [10,11,12]. Third, the parameter $S_{2}^{i,j}$ represents the pixelwise, temperature-dependent NU noise produced by power dissipation and changes in the FPA’s surrounding temperature. Fourth, it can be noticed that, when temperature changes at the FPA are negligible, the model reduces to the classical temperature-independent, first-order model for the NU noise. Lastly, the temperature changes at the FPA cannot be easily estimated: Typical temperature sensors in IR cameras have relatively low resolution, and fast changes in the surrounding temperature produce differences between the sensor readings and the actual temperature of the FPA.

### 2.2. Compensation Model, Parameter Estimators, and Temperature Variation Tracking Estimator

To estimate the true incident radiation ${\hat{X}}^{i,j}\left[k\right]$, at the (i,j)th pixel and at the *k*th sample time, we propose the following linear estimator:

(6) $$\begin{array}{c} {\hat{X}}^{i,j}\left[k\right]={\hat{S}}_{0}^{i,j}Y^{i,j}\left[k\right]+{\hat{S}}_{1}^{i,j}+{\hat{S}}_{2}^{i,j}\hat{\Delta T}\left[k\right]+\Omega \left(\Delta T\left[k\right]\right) \end{array}$$

where ${\hat{S}}_{k}^{i,j}$ is an estimate of the NU noise parameter $S_{l}^{i,j}$ (for l=0,1,2), $\hat{\Delta T}\left[k\right]$ is an online estimate of the FPA temperature changes with respect to a reference temperature measured at k=0, and Ω(ΔT[k]) is an online estimator for $X_{h}\left[k\right]$ and any unmodeled nonlinear dynamics. For simplicity, we state two assumptions: (i) the temperature variations are spatially uniform on the FPA; and (ii) at k=0, the IR irradiance generated in the housing of the IR camera is negligible.

We propose to estimate first the three NU noise parameters $S_{l}^{i,j}$ in Equation (5) using a calibration-based approach. To do so, starting at k=0, we place the IR camera in front of a blackbody radiator at three known temperatures, say, $X_{1}$, $X_{2}$, and $X_{3}$. Concurrently, we induce a temperature change inside of the camera and measure the temperature differences, $\Delta T_{1}\left[0\right]$, $\Delta T_{2}\left[0\right]$, and $\Delta T_{3}\left[0\right]$, using an embedded sensor. Consequently, we can compute estimates for the NU noise parameters solving the pixelwise system of equations:

(7) $$\begin{array}{cc} {\hat{S}}_{0}^{i,j}Y_{1}^{i,j}\left[0\right]-{\hat{S}}_{1}^{i,j}-{\hat{S}}_{2}^{i,j}\Delta T_{1}\left[0\right] & =X_{1}^{i,j}\left[0\right] \end{array}$$

(8) $$\begin{array}{cc} {\hat{S}}_{0}^{i,j}Y_{2}^{i,j}\left[0\right]-{\hat{S}}_{1}^{i,j}-{\hat{S}}_{2}^{i,j}\Delta T_{2}\left[0\right] & =X_{2}^{i,j}\left[0\right] \end{array}$$

(9) $$\begin{array}{cc} {\hat{S}}_{0}^{i,j}Y_{3}^{i,j}\left[0\right]-{\hat{S}}_{1}^{i,j}-{\hat{S}}_{2}^{i,j}\Delta T_{3}\left[0\right] & =X_{3}^{i,j}\left[0\right] \end{array}$$

We note that several researchers, including our group, have observed that the temperature changes at the FPA exhibit a non-linear behavior with respect to the raw output [8,15,16,17,18,19]. Thus, to compensate for both the aforementioned non-linear effect and any other unmodeled dynamics associated with the term $X_{h}\left[k\right]$ (i.e., the temperature changes at the FPA), we employ a HW estimator for the spatially uniform term Ω(ΔT[k]). The HW estimator, which is a block-oriented combining linear dynamic models with nonlinear functions, is computed offline following the standard procedure described in [22], where the non-linear input channel required by the estimator corresponds to samples of the FPA temperature sensor and the Model (5) is the demanded linear function [22].

To avoid the limitations of low-resolution temperature sensors, we estimate the FPA temperature changes online from the statistics of the scene. The estimation model assumes that: (i) the irradiance generated in the IR camera is negligible; and (ii) the FPA temperature is a short-term constant which can be estimated using a recursive LMS approach. More precisely, if we minimize the total squared error between the estimated incident IR irradiance, ${\hat{X}}^{i,j}\left[k\right]$, and its local spatial average, $E[{\hat{X}}^{i,j}\left[k\right]]$, it is straightforward to show that the recursive LMS estimator is:

(10) $$\begin{array}{cc} \hat{\Delta T}\left[k\right] & =\hat{\Delta T}\left[k-1\right]+2\eta \sum_{i=1}^{M}\sum_{j=1}^{N}{\hat{S}}_{2}^{i,j}\left({\hat{X}}^{i,j}\left[k-1\right]-E\left[{\hat{X}}^{i,j}\left[k-1\right]\right]\right) \end{array}$$

where *η* is the LMS learning rate and the local spatial average, at the (i,j)th pixel, can be computed using the formula:

(11) $$\begin{array}{c} E\left[{\hat{X}}^{i,j}\left[k\right]\right]=\frac{1}{{\left(2W+1\right)}^{2}}\sum_{m=i-W}^{i+W}\sum_{n=j-W}^{j+W}{\hat{X}}^{n,m}\left[k\right] \end{array}$$

with (2W+1)×(2W+1) the size of the spatial averaging window.

### 2.3. Experimental Setup

In order to test the efficiency of the proposed model and compensation algorithm, we experimentally obtained a set of real data from two microbolometer-based cameras and a calibrated IR source. We used a FLIR Tau 2 IR core with a 640×512-pixel vanadium-oxide FPA operating in the 7.5μm–13.5μm range at 30 frames per second (fps). The camera quantizes the impinging IR radiation using 14 bits. We also used a CEDIP Jade UR IR camera with a 320×240-pixel amorphous silicon FPA, with a pitch size of 40 μm, operating between 8μm and 12μm at 60 fps. The camera quantizes the IR radiation using 12 bits, includes 25 mm 1/f optics, and it has a germanium window in front of the FPA. The IR source is a black-body calibrator Mikron M345 from LumaSense Technologies. The black-body source provides a large emission area (12”×12”) with an emissivity of 0.9756 ± 0.0039 in the 8 to 15 μm spectral band, at temperatures in a range from 0 ${}^{\circ }$C to 150 ${}^{\circ }$C and with an accuracy of ±0.05 ${}^{\circ }$C.

The calibration setup uses the black-body radiator set at different temperatures to obtain the NUC parameters and the temperature-dependent term. We performed the calibration in a temperature-controlled room by focusing the IR camera on two black-body radiators, while controlling the room temperature as well as the surrounding temperature of the camera. In order to obtain the NUC parameters, two black-body temperature points are the required, at minimum, as stated in the calibration procedure (7)–(9). To produce the required temperature variations in the array of microbolometers, we heated the surroundings of the camera using warm air, and measured the FPA temperature using the camera controlling software.

### 2.4. Performance Metrics

To test the performance of the NUC proposed in this paper we employ the following performance metrics. First, as a quantitative measure of NUC, we use the root mean square error (RMSE), averaged over all the pixels in an image, between a reference IR image and either a raw or a NU compensated image. The RMSE, at the *n*th IR image, is calculated using:

(12) $$\mathrm{RMSE}\left(n\right)={\left(\frac{1}{pq}\sum_{i=1}^{p}\sum_{j=1}^{p}{\left({\hat{x}}_{i,j}\left(n\right)-x_{i,j}\left(n\right)\right)}^{2}\right)}^{1/2}$$

where p×m is the number of pixels in the FPA, ${\hat{x}}_{i,j}\left(n\right)$ is the IR irradiance obtained after the NUC at the ijth pixel of the image, and $x_{i,j}\left(n\right)$ is the reference IR irradiance, at the ijth pixel, calculated using black-body radiator data.

Second, for cases where a reference frame is not available, we use as a performance metric the so-called roughness index, denoted as *ρ*, to estimate the quality of the NUC [11,12,13,14]. The roughness index of the *n*th image is calculated as:

(13) $$\rho \left(X\left(n\right)\right)={∥X\left(n\right)∥}_{2}^{-1}\left(∥h_{1}{⊗X\left(n\right)∥}_{2}+{∥h_{1}^{T}⊗X\left(n\right)∥}_{2}\right)$$

where $h_{1}$ is the 1×2 spatial filter [-11], *X* is an image, ${\left||X\left(n\right)|\right|}_{2}$ is the $\ell_{2}$ norm of X(n) (Euclidean norm) and ⊗ represents the discrete 2D circular convolution. Note that *ρ* is zero for an uniform image and increases with detector-to-detector variations.

Lastly, and also to estimate the quality of the images rendered by the proposed algorithm after the NUC, we use the cosine similarity ($C_{s}$) [23]. The cosine similarity computes the normalized inner product between two vectors, and thus measures the similarity between the vectors independently of their magnitudes. Therefore, this metric is suitable to assess the similarity between two images with different global intensities, which is the typical result of scene-based NUC methods that are not radiometrically accurate. The cosine similarity is given by the following equation:

(14) $$C_{s}\left(X\left(n\right),\hat{X}\left(n\right)\right)=\frac{\sum_{i=1}^{p}\sum_{j=1}^{q}x_{i,j}\left(n\right){\hat{x}}_{i,j}\left(n\right)}{\sqrt{\sum_{i=1}^{p}\sum_{j=1}^{q}x_{i,j}\left(n\right)x_{i,j}\left(n\right)}\sqrt{\sum_{i=1}^{p}\sum_{j=1}^{q}{\hat{x}}_{i,j}\left(n\right){\hat{x}}_{i,j}\left(n\right)}}$$

where $\hat{X}\left(n\right)$ is the *n*th NU noise-compensated image of size p×q pixels, whose ijth pixel is denoted as ${\hat{x}}_{i,j}\left(n\right)$, and X(n) is the *n*th reference image obtained from an IR camera after two-point calibration using a blackbody radiator, with ijth pixel denoted as $x_{i,j}\left(n\right)$. When both vectors are aligned, $C_{s}\left(X\left(n\right),\hat{X}\left(n\right)\right)$ is equal to 1. If the vector representations of the images are orthogonal, their similarity is equal to 0.

## 3. Results and Discussion

We performed the calibration in Equations (7)–(9) with an initial FPA temperature of 26 ${}^{\circ }$C for the FLIR core and 34 ${}^{\circ }$C for the CEDIP camera, $\Delta T_{1}\left[0\right]=\Delta T_{2}\left[0\right]=0{\;}^{\circ }$C, $\Delta T_{3}\left[0\right]=4{\;}^{\circ }$C, and set a black-body radiator at $X_{1}^{i,j}\left[0\right]=20{\;}^{\circ }$C and $X_{2}^{i,j}\left[0\right]=X_{3}^{i,j}\left[0\right]=30{\;}^{\circ }$C. We designed a third-order HW estimator using FPA temperature samples between $11{\;}^{\circ }$C and $38{\;}^{\circ }$C. All model parameters were obtained 6 months before the experiments presented below.

**Figure 2:**
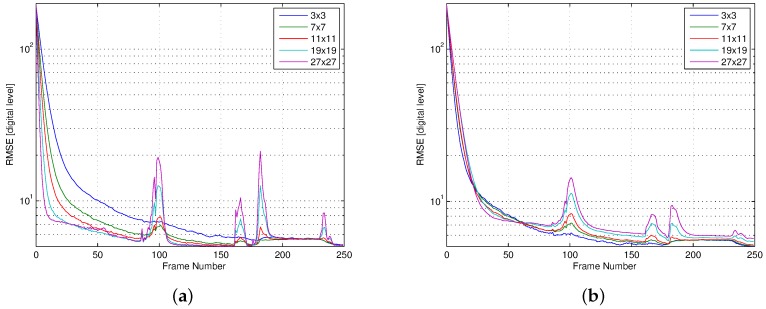
The root mean square error (RMSE) between a reference image and a non-uniformity (NU) compensated image using the proposed method, as a function of the frame number, and for different values of the window size. (**a**) The same learning rate was used for all window sizes; (**b**) The learning rate was adjusted, for each window size, to obtain a similar settling time.

In order to select the learning rate *η* and window size *W*, we used the FLIR core to capture IR video in a room, and calibrated the output using black-body radiators. We run our algorithm on the raw output of the core to estimate $X^{i,j}\left[k\right]$ using Equation (6), and computed the RMSE between our estimation and the calibrated output. The online estimation of $\hat{\Delta T}\left[k\right]$ is initially performed on each video frame, while the HW estimator runs every 500 ms. The FPA temperature remained constant during the experiment. Figure 2 shows the RMSE as a function of the video frame number for different-size windows.

**Figure 3:**
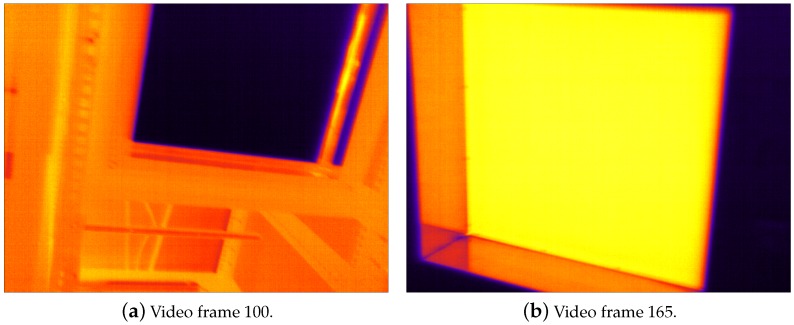
Video frames that introduce large spatial gradients.

In the graph of Figure 2a, we used the same learning rate independently of the window size. Because larger windows can capture spatial temperature gradients in the scene, the value of the error term ${\hat{X}}^{i,j}\left[k-1\right]-E\left({\hat{X}}^{i,j}\left[k-1\right]\right)$ in Equation (10) may be larger for these windows. Consequently, using larger windows results in a shorter settling time than smaller windows. The graph also shows RMSE peaks around video frame numbers 100, 165, 185, and 235. These peaks occur when objects that produce large spatial gradients enter the scene (e.g., cold surfaces and black-body radiators), as shown in Figure 3. These gradients generate a larger error term, causing an increase in the RMSE. This effect is significantly more pronounced for larger window sizes. Figure 2b repeats the experiment, this time adjusting the learning rates empirically to achieve a similar settling time for different window sizes. The graph shows that, after 60 video frames (2 s), the RMSE settles to values below 5 digital counts, which is equivalent to $0.14{\;}^{\circ }$C. For a 3×3-pixel window, the RMSE remains less than 2 digital counts ($0.06{\;}^{\circ }$C) after 100 video frames, and it exhibits peaks of almost negligible magnitude. In consequence, we used a 3×3-pixel window (W=1) and a learning rate η=0.0012 in the rest of our experiments.

**Figure 4:**
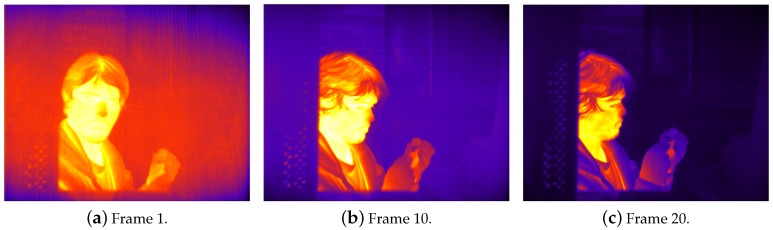
Model output on FLIR core at startup, T${}_{FPA}=22{\;}^{\circ }$C.

Figure 4 shows three video frames from the FLIR core after starting the algorithm with the FPA temperature $T_{FPA}=22{\;}^{\circ }$C. After only 20 iterations (670 ms), the algorithm successfully removes all visible NU artifacts from the image. The recursion in Equation (10) converges in less than 100 video frames (3.3 s). Because changes in the FPA temperature are typically slow, we run the estimator every 500 ms after this initial sequence, thus greatly reducing the computational requirements of the algorithm.

**Figure 5:**
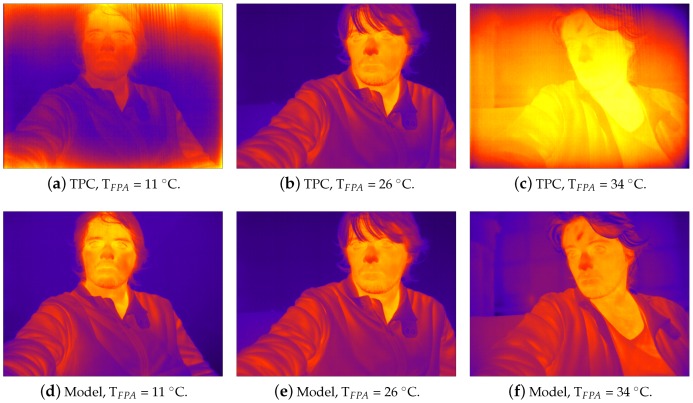
Video captures from the FLIR core at different focal plane array (FPA) temperatures. (**top**) Using two-point calibration (TPC); (**bottom**) Using our model.

Figure 5 compares the performance of regular two-point calibration (TPC) to our model correcting visual NU artifacts in the presence of changes in $T_{FPA}$. Both the model and TPC parameters were computed with $T_{FPA}=26{\;}^{\circ }$C, and the images were captured from the FLIR core while we heated the environment to vary $T_{FPA}$ from $11{\;}^{\circ }$C to $34{\;}^{\circ }$C. TPC successfully corrects the image only when the temperature of the FPA is the same used for calibration, see Figure 5b. For other temperatures, the image exhibits NU artifacts induced by the FPA and imaging optics [24]. Unlike TPC, our algorithm successfully eliminates such artifacts not only at $T_{FPA}=26{\;}^{\circ }$C, but also for all values.

Videos attached as Supplementary Material show both algorithms operating in real time at $T_{FPA}=29{\;}^{\circ }$C. Video 1 is the raw output of the camera, showing severe FPN artifacts. Video 2 is the output produced by regular TPC when the calibration was performed at the same temperature of $29{\;}^{\circ }$C. At this operating temperature, the algorithm successfully corrects the artifacts present in the raw video. Video 3 is the output of the same algorithm, but with parameters obtained at $26{\;}^{\circ }$C. The difference between calibration and operation temperature produces noticeable FPN artifacts from the FPA and the camera optics. In contrast, Video 4 shows the output produced by the method proposed in this paper. Despite using parameters obtained at $26{\;}^{\circ }$C and operating at $29{\;}^{\circ }$C, the resulting image is free of FPN artifacts.

**Figure 6:**
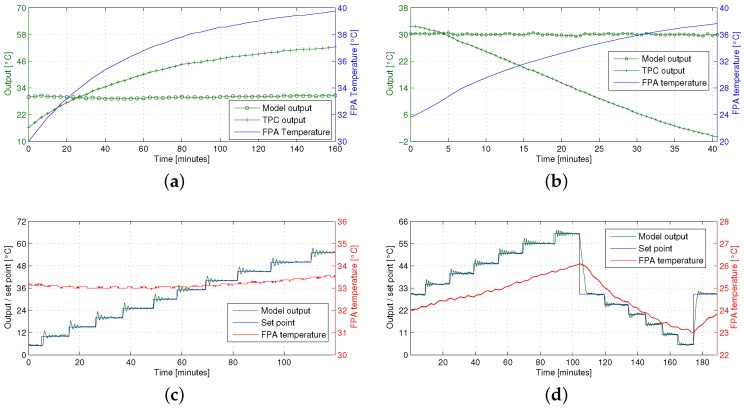
Model output with varying FPA and black-body temperature. (**a**) Model and TCP output with varying T${}_{FPA}$, CEDIP camera; (**b**) Model and TCP output with varying T${}_{FPA}$, FLIR core; (**c**) Model output with varying black-body temperature, CEDIP camera; (**d**) Model output with varying black-body temperature, FLIR core.

To evaluate the performance of our algorithm quantitatively, we used the camera to measure the temperature of a black-body radiator, using a polynomial fit to map the digital counts from the camera to temperature values. The black body temperature, $T_{BB}$, was set to 30 ${}^{\circ }$C, and we progressively heated the room to vary $T_{FPA}$. Figure 6a shows the results for the CEDIP camera, where $T_{FPA}$ increased by $10{\;}^{\circ }$C during the experiment. Using TPC, the estimate of $T_{BB}$ was correct only when $T_{FPA}=34{\;}^{\circ }$C, which corresponds to the FPA temperature when the TPC was carried out. Otherwise, the estimate of $T_{BB}$ varied from $31{\;}^{\circ }$C to $37{\;}^{\circ }$C. The output of our model remained stable at 30 ${}^{\circ }$C, with an average RMSE of $0.22{\;}^{\circ }$C and a median RMSE of $0.27{\;}^{\circ }$C throughout the experiment. Figure 6b shows the same results for the FLIR core, with a $T_{FPA}$ increase of $14{\;}^{\circ }$C in 40 min. Again, the TPC estimate varies by $33{\;}^{\circ }$C and is correct only when the FPA is at its calibration temperature of $26{\;}^{\circ }$C. Our model correctly estimates $T_{BB}=30{\;}^{\circ }$C, with an average RMSE of $0.25{\;}^{\circ }$C and a median RMSE of $0.24{\;}^{\circ }$C.

Figure 6c illustrates another experiment where we varied the set point of the black-body radiator from $5{\;}^{\circ }$C to $55{\;}^{\circ }$C in $5{\;}^{\circ }$C steps, and plotted our estimate of $T_{BB}$ using the CEDIP camera. The temperature of the FPA varied between $33{\;}^{\circ }$C and $33.5{\;}^{\circ }$C during the experiment, mainly due to the changes in environmental temperature induced by the black body. The ringing in the model output is due to the black-body temperature control loop, as confirmed by its own temperature readings. The model provides an accurate estimate of $T_{BB}$ throughout the black-body temperature range. Figure 6c also illustrates the coarse quantization provided by the embedded temperature sensor. Figure 6d shows another experiment, where we used the FLIR core and varied the black-body set point between $5{\;}^{\circ }$C and $60{\;}^{\circ }$C. The FPA temperature of the IR core is notably more sensitive to changes in the environment than the CEDIP camera, varying between $23{\;}^{\circ }$C and $26{\;}^{\circ }$C. Again, the model provides an accurate online estimate of $T_{BB}$ throughout the entire experiment.

Next, we assess the quality of the images rendered by the proposed NUC method in real IR video sequences. In addition, we compare our NUC method with the following scene-based methods: the Enhanced Constant Range (ECR) NUC method [25], Scribner’s retina-like NUC method [26], and a Kalman filter-based NUC method [12]. To do so, we use the roughness metric and the cosine similarity between the output of each method and a TPC-corrected image. Please note that, because we calibrated the camera and captured the IR videos at the same FPA operating temperature, we can use the TPC method as the benchmark for the comparison.

**Figure 7:**
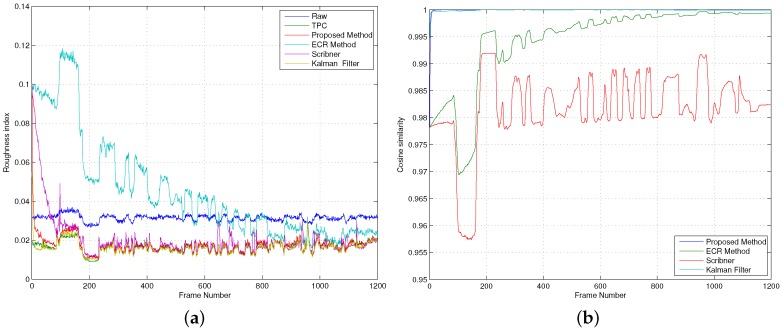
Assessment of the image quality rendered by the different non-uniformity correction (NUC) methods. (**a**) Roughness index; (**b**) Cosine similarity.

Figure 7 shows the roughness and the cosine similarity metrics for all the NUC methods. The best performance, for both metrics, is achieved by the proposed method and the Kalman filter, followed by Scribner’s method and the ECR method. The best performance of the former two algorithms is attributed to their prediction/update estimation approach. Kalman filters are well-known by their prediction/update scheme. The prediction step of the proposed method is carried out during the estimation of the NU parameters and the HW model, and the update step is carried out using the raw output and past estimates. The Scribner and the ECR methods are simpler because they lack a prediction step.

**Figure 8:**
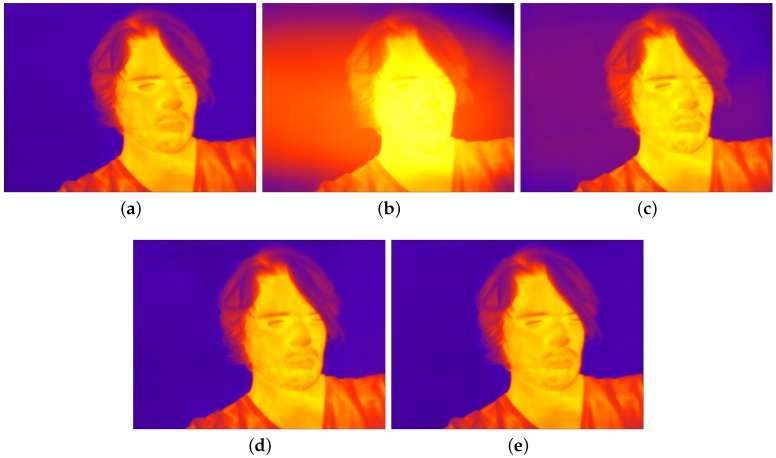
(**a**) Sample raw frame taken from a sequence of IR data. NU compensated versions of the raw frame obtained using: (**b**) Scribner’s retina-like method; (**c**) Enhanced Constant Range method; (**d**) Kalman filter NUC method; and (**e**) Proposed method.

Figure 7a depicts the roughness metric, as a function of the frame number, for all the NUC methods. We also plot the roughness metric of the raw video as a reference. It can be observed from the figure that all the NUC methods effectively compensate for the NU noise because their roughness metrics are lower than the roughness metric of the raw video. Note that both the proposed method and the Kalman filter exhibit the lowest roughness metric, which is almost equal to the roughness metric exhibited by the TPC method. Moreover, such excellent NUC capability is achieved after only 100 video frames (3.3 s), approximately. Figure 7b shows the cosine similarity metric, as a function of the frame number, for all the NUC methods. It can be observed that all the NUC methods render compensated images that are very similar to those obtained by a TPC method. According to the cosine similarity, metric the NUC capability of the proposed algorithm is not only the best, but it is also achieved even faster than in the case of the roughness metric: It takes only 10 frames (0.33 s) to obtain a cosine similarity of 0.9997. We also note that the results of the two performance metrics are supported by the sample images taken from the IR video sequence shown in Figure 8. It can be noticed that, as in Figure 4, the proposed algorithm does not introduce NUC artifacts in the rendered images. Lastly, we highlight that the proposed method not only compensates for the NU noise but also produces a temperature-calibrated output, while the three scene-based NUC methods can only produce NUC images.

Finally, in Table 1 we list the parameters and the number of offline and online calculations performed by each NUC algorithm used in this paper. In this implementation of our method, W=1 and n=3. Table 1 shows that, unlike the other three algorithms, the proposed method requires the calculation of an additional NUC parameter and the estimation of the HW model. Such calculations, however, are performed offline, do not demand heavy computations, and are not carried out in a pixelwise fashion. From Table 1, it can be observed that the proposed algorithm imposes an online workload almost equal to the workload required by Scribner’s NUC method. In fact, for the 3×3 pixel window used here, only 9 more additions must be carried out compared to the Kalman filter method.

## 4. Conclusions

The model and algorithm presented in this paper provides online NUC for uncooled microbolometer-based IR cameras. The model separately estimates the NU noise introduced by constant fabrication-dependent parameters, and temperature-dependent components with fixed structure. A combination of initial calibration and online estimation results in accurate estimation of the subject temperature. Although we use scene statistics to estimate the FPA temperature, the fixed structure of the noise prevents ghosting artifacts, a well-known limitation of scene-based NUC methods. Our experiments have shown that we can compensate the output of IR cameras with a RMSE of less than 0.25${\;}^{\circ }$C, when the temperature of the FPA varies approximately in 15${\;}^{\circ }$C. In addition, results shown here indicate that large estimation errors in the input temperature may be induced if no compensation or calibration is employed. Thus, providing mechanisms for compensating the camera output, while avoiding frequent calibrations, is an appealing feature of our NUC method.

We are currently working on implementing the algorithm in embedded hardware/software with the long-term goal of developing intelligent, autonomous, and inexpensive infrared visualization systems, which provide real-time operation with low power consumption on embedded hardware.

## Figures and Tables

**Table 1:** Parameters and calculations perfomed by each NUC algorithm. (2W+1)×(2W+1) is the window size, and *n* is the order of the Hammerstein-Wiener (HW) estimator.

	Compute			Offline	Online Calculations (per pixel)		
Algorithm	Gain	Offset	Other	Calculations	Additions	Multiplications	Other
Proposed	Yes	Yes	HW model & thermal offset	Solve 3×3 and n×n linear eqs.	${\left(2W+1\right)}^{2}+5$	5	—
ECR	Yes	Yes	No	—	5	5	1 abs. value 1 comparison
Kalman	No	Yes	No	—	5	5	—
Scribner	Yes	Yes	No	—	${\left(2W+1\right)}^{2}$ + 4	3	—

## Supplementary Materials

The following videos are available online at https://zenodo.org/record/58163: Video 1 is the raw output of the camera; Video 2 and Video 3 are the output produced by regular TPC at $29{\;}^{\circ }$C and $26{\;}^{\circ }$C, respectively; and Video 4 is the output produced by the method proposed in this article.

## Abbreviations

The following abbreviations are used in this manuscript:

FPA	focal plane array
FPN	fixed-pattern noise
IR	infrared
HW	Hammerstein-Wiener
LMS	least mean squares
LWIR	long-wave infrared
NUC	non-uniformity correction
NU	non-uniformity
TPC	two-point calibration
TRC	thermal resistance coefficient
MPC	multi-point calibration
fps	frames per second
RMSE	root mean square error
ECR	Enhanced Constant Range

## References

[1] Wood R.A. (1995). Camera for Producing Video Output Signal, Infrared Focal Plane Array Package for such Camera, and Method and Apparatus for Generating Video Signals from Passive Focal Plane Array of Elements on a Semiconductor Substrate. U.S. Patent.

[2] Rogalski A. (2011). Infrared Detectors.

[3] Holst G.C. (1998). CCD Arrays, Cameras, and Displays.

[4] Mooney J.M., Sheppard F.D., Ewing W.S., Ewing J.E., Silverman J. (1989). Responsivity Nonuniformity Limited Performance of Infrared Staring Cameras. Opt. Eng..

[5] Thomas P., Sinclair P., Savachenko A., Goldman P., Elinas P., Pope T. Signal calibration and stability in an uncooled integrated bolometer array. Proceedings of the IEEE Aerospace Conference.

[6] Bieszczad G., Orżanowski T., Sosnowski T., Kastek M. Method of detectors offset correction in thermovision camera with uncooled microbolometric focal plane array. Proceedings of the Electro-Optical and Infrared Systems: Technology and Applications VI, 74810O.

[7] Nugent P.W., Shaw J.A., Pust N.J. (2013). Correcting for focal-plane-array temperature dependence in microbolometer infrared cameras lacking thermal stabilization. Opt. Eng..

[8] Cao Y., Tisse C.L. (2013). Shutterless solution for simultaneous focal plane array temperature estimation and nonuniformity correction in uncooled long-wave infrared camera. Appl. Opt..

[9] Perry D.L., Dereniak E.L. (1993). Linear theory of nonuniformity correction in infrared staring sensors. Opt. Eng..

[10] Narendra P.M. (1982). Scene-based nonuniformity compensation for imaging sensors. IEEE Trans. Pattern Anal. Mach. Intell..

[11] Harris J.G., Chiang Y.M. (1999). Nonuniformity Correction of Infrared Image Sequences Using the Constant-Statistics Constraint. Trans. Image Process..

[12] Torres S.N., Hayat M.M. (2003). Kalman filtering for adaptive nonuniformity correction in infrared focal-plane arrays. J. Opt. Soc. Am. A.

[13] Martin C.S., Torres S., Pezoa J.E. (2008). Statistical recursive filtering for offset nonuniformity estimation in infrared focal-plane-array sensors. Infrared Phys. Technol..

[14] Vera E., Meza P., Torres S. (2011). Total variation approach for adaptive nonuniformity correction in focal-plane arrays. Opt. Lett..

[15] Nugent P.W., Shaw J.A., Pust N.J. (2013). Correcting for focal-plane-array temperature dependence in microbolometer infrared cameras lacking thermal stabilization. Opt. Eng..

[16] Krupiński M., Bieszczad G., Sosnowski T., Madura H., Gogler S. (2014). Non-Uniformity Correction in Microbolometer Array with Temperature Influence Compensation. Metrol. Meas. Syst..

[17] Krupiński M., Bieszczad G., Gogler S., Madura H. Non-uniformity correction with temperature influence compensation in microbolometer detector. Proceedings of the Image Sensing Technologies: Materials, Devices, Systems, and Applications II.

[18] Pedreros F., Pezoa J.E., Torres S.N. Compensating internal temperature effects in uncooled microbolometer-based infrared cameras. Proceedings of the Infrared Imaging Systems: Design, Analysis, Modeling, and Testing XXIII, Baltimore Inner Harbor.

[19] Wolf A., Redlich R., Figueroa M., Pezoa J.E. On-line nonuniformity and temperature compensation of uncooled IRFPAs using embedded digital hardware. Proceedings of the Infrared Sensors, Devices, and Applications III.

[20] Olbrycht R., Więcek B. (2015). New approach to thermal drift correction in microbolometer thermal cameras. Quant. InfraRed Thermogr. J..

[21] Daniels A. (2010). Field Guide to Infrared Systems, Detectors, and FPAs.

[22] Bai E.W. (1998). An optimal two-stage identification algorithm for Hammerstein—Wiener nonlinear systems. Automatica.

[23] Tan P.N., Steinbach M., Kumar V. (2005). Introduction to Data Mining.

[24] Cao Y., Tisse C.L. (2014). Single-image-based solution for optics temperature-dependent nonuniformity correction in an uncooled long-wave infrared camera. Opt. Lett..

[25] Pezoa J.E., Torres S.N., Córdova J.P., Reeves R.A. (2004). An Enhancement to the Constant Range Method for Nonuniformity Correction of Infrared Image Sequences. Progress in Pattern Recognition, Image Analysis and Applications.

[26] Scribner D., Sarkady K.A., Kruer M., Caulfield J., Hunt J.D., Colbert M., Descour M. Adaptive retina-like preprocessing for imaging detector arrays. Proceedings of the IEEE International Conference on Neural Networks.

